# Dosimetric comparison of linear accelerator-based stereotactic radiosurgery systems

**DOI:** 10.4103/0971-6203.31145

**Published:** 2007

**Authors:** S. D. Sharma, Sudhir Kumar, S. S. Dagaonkar, Geetika Bisht, S. Dayanand, Reena Devi, S. S. Deshpande, S. Chaudhary, B. C. Bhatt, S. Kannan

**Affiliations:** Radiological Physics and Advisory Division, Bhabha Atomic Research Centre, CT and CRS Building, Anushaktinagar, Mumbai - 400 094, India; * Radiation Safety Systems Division, Bhabha Atomic Research Centre, Trombay - 400 085, India; # Bombay Hospital, Marine Lines, Mumbai - 400020, India; ^ Department of Medical Physics, Tata Memorial Hospital, Mumbai - 400 012, India; † Advanced Centre for Treatment Education and Research in Cancer, Navi Mumbai, India; ‡ P. D. Hinduja National Hospital, Mahim, Mumbai - 400 016, India; § CSIR/BARC, CT and CRS Building, Anushaktinagar, Mumbai - 400 094, India

**Keywords:** Circular cones, dosimetry, micro multileaf collimator, radiosurgery, X-knife

## Abstract

Stereotactic radiosurgery (SRS) is a special radiotherapy technique used to irradiate intracranial lesions by 3-D arrangements of narrow photon beams eliminating the needs of invasive surgery. Three different tertiary collimators, namely BrainLab and Radionics circular cones and BrainLab micro multileaf collimator (mMLC), are used for linear accelerator-based SRS systems (X-Knife). Output factor (S_t_), tissue maximum ratio (TMR) and off axis ratio (OAR) of these three SRS systems were measured using CC01 (Scanditronix/ Welhofer) and Pinpoint (PTW) cylindrical and Markus plane parallel ionization chambers as well as TLD and radiochromic film. Measurement results of CC01 and Pinpoint chambers were very close to each other which indicate that further reduction in volume and physical dimensions of cylindrical ionization chamber is not necessary for SRS/SRT dosimetry. Output factors of BrainLab and Radionics SRS cones were very close to each other while output factors of equivalent diameter mMLC field were different from SRS circular cones. TMR of the three SRS systems compared were very close to one another. OAR of Radionics cone and BrainLab mMLC were very close to each other, within 2%. However, OARs of BrainLab cone were found comparable to OARs of Radionics cone and BrainLab mMLC within maximum variation of 4%. In addition, user-measured similar data of other three mMLC X-Knives were compared with the mMLC X-Knife data measured in this work and found comparable. The concept of switching over to mMLC-based SRS/SRT is thus validated from dosimetric characteristics as well.

## Introduction

Stereotactic radiosurgery (SRS) is a special radiotherapy technique used to irradiate intracranial lesions by means of three-dimensional arrangements of narrow photon beams eliminating the needs of invasive surgery.[1] In SRS, high radiation dose (10-24 Gy) is given in a single fraction in contrast to conventional fractionated radiotherapy or fractionated stereotactic radiotherapy (SRT). SRS of intracranial lesion combines the use of a stereotactic apparatus and energetic radiation beams to irradiate the lesion with a single treatment while SRT utilizes the stereotactic apparatus and radiation beams for multiple treatments.[2] By these treatment techniques, a concentrated dose is delivered to the lesion with steep dose gradients external to the treatment volume. The rapid dose fall-off from the edge of the treatment volume provides dramatic sparing of normal brain tissues.

SRS is used to manage several diseases such as arteriovenous malformations (AVM), benign and malignant brain tumors and functional disorders.[1–5] Heavy charged particles (e.g., protons), gamma rays and megavoltage X-rays have been used to irradiate arteriovenous malformations as well as benign and malignant tumors. However, in India, only gamma rays from a dedicated treatment unit (Leksell Gamma Knife) and megavoltage X-rays from medical linear accelerators are in use at a number of centers in the country to perform such a high-precision technique using external photon beams. Medical linear accelerator-based radiosurgery system (X-Knife) uses either narrow circular cones or micro multileaf collimator (mMLC) to shape the treatment fields. Circular X-Knife collimators of two different manufacturers namely, BrainLab and Radionics and mMLC of BrainLab are used commonly in India for SRS/SRT. The design and construction material of these tertiary collimators used to shape X-Knife fields are different from one another which may result in dissimilar dosimetric characteristics.[6–9] Dosimetric characteristics of these three different X-Knife collimators were determined using a single dosimeter under similar experimental conditions and compared.

The small dimension and steep dose gradient of photon beams used in SRS/SRT require an appropriate dosimeter with spatial resolution less than 1.0 mm. Due to small beam diameter, there is a lack of lateral electronic equilibrium which further complicates the dosimetry.[10–12] The relative sensitivity of the dosimeter used should be independent of dose rate and energy in order to give a linear dose response throughout the measured dose profiles and dose distributions. An ideal detector should be water equivalent to cause as little interference as possible with the radiation field.[13] The SRS/SRT dosimetry techniques using different detectors and related problems have been extensively reported.[9,14–20]

To fulfill the requirements for dosimetry of narrow SRS/SRT photon fields, a number of new miniature (i.e., high resolution) detectors of different types [ionization chambers, diodes, films, diamond detectors, thermoluminescent (TL), etc.] have been introduced commercially. Among these detectors, miniature ionization chambers are commonly used because of their obvious superiority (redundancy, convenience, cost, availability, etc.) over other detector systems. A new miniature cylindrical ionization chamber (CC01, Scanditrinix/Welhofer Dosimetrie, Sweden) of comparatively smaller volume has been brought in commercially with the intention to eliminate the problems associated with ionization chamber dosimetry of SRS/SRT beams. Dosimetric measurements on X-Knives narrow beams were therefore carried out using CC01 and other suitable detectors to verify its relative suitability for dosimetry of small SRS/SRT fields.

## Materials and Methods

### X-Knives

Dosimetric measurements were carried out on three different X-Knives installed at three different radiotherapy centers. These three X-Knives were using Siemens Primus medical linear accelerators (Linac) for generating X-ray beams. The first Linac was using BrainLab circular cones (cone diameter in the range of 7.5-50 mm), second Linac was using Radionics circular cones (cone diameter in the range of 12.5-50 mm) and the third Linac was using BrainLab m3 mMLC as tertiary collimators. BrainLab circular cones are made up of lead in brass shell (length = 11.5 cm, outer diameter = 10.8 cm) and Radionics circular cones are made up of cerrobend in stainless steel shell (length = 12.5 cm, outer diameter = 7.5 cm). BrainLab m3 mMLC has 52 (26 pairs) tungsten leaves which move perpendicular to the beam central axis. Central 14 leaf pairs of m3 have finer leaf width of 3 mm at isocentre which allow improved beam shaping around targets usually treated by radiosurgery. However, peripheral leaf pairs of m3 are of width 4.5 (6 × 4.5 mm: three each to the left and right of 3 mm leaves) and 5.5 mm (6 × 5.5 mm: three each to the left and right of 4.5 mm leaves) at isocentre, respectively. The maximum square field area that can be defined at isocentre is 10.2 × 10 cm^2^. The specified positional accuracy and reproducibility of the leaf are better than 0.1 mm. All the measurements were carried out with 6MV X-ray beams. A constant 6×6 cm^2^ secondary collimator (jaw/ conventional MLC) field was kept open during all the measurements.

### Dosimeters

Two miniature cylindrical ionization chambers, namely Compact (CC01, Scanditronix/Wellhofer Dosimetrie, Sweden) and Pinpoint (PTW Freiburg, Germany); a plane parallel (PP) ionization chamber (Markus, PTW Freiburg, Germany), TLD-100 powder and radiochromic film (Gafchromic MD-55-2, ISP Technology, USA) were used for the dosimetry measurements on X-Knives. Technical details of ionization chambers used for measurements on SRS/SRT systems are listed in Table 1.

**Table 1:** Technical specifications of ionization chambers used for measurements on stereotactic radiosurgery/ stereotactic radiotherapy systems

*Parameters*	*Compact cylindrical chamber*	*Pinpoint cylindrical chamber*	*Markus PP chamber*
Active volume	0.012 cm^3^	0.015 cm^3^	0.055 cm^3^
Active length/diameter	3.6 mm	5.0 mm	5.3 mm
Inner diameter/plate separation	2.0 mm	3.7 mm	2.0 mm
Wall/front window thickness	0.5 mm	0.71 mm	0.03 mm
Wall/front window material	Shonka C552	PMMA (r = 1.19 g/cm^3^) + Graphite	Polyethylene + graphite
	(ρ = 1.76 g/cm^3^)	(ρ = 0.82g/cm^3^)	(ρ = 0.82 g/cm^3^)
Material of inner/collecting electrode	Steel	Steel	Acrylic + graphite
	(ρ = 8.5 g/cm^3^)		(ρ = 0.82 g/cm^3^)
Diameter of inner/collecting electrode	0.35 mm	0.18 mm	5.3 mm
Leakage current	< ± 7.5 × 10^−16^ A	< ± 4 × 10^−15^ A	< ± 2 × 10^−16^ A
Sensitivity	3.3 × 10^−10^ C/Gy	4 × 10^−10^ C/Gy	2 × 10^−9^ C/Gy

Radiochromic film MD-55-2 is a double sensitive layer dispersion coated film. The sensitive layer of the film changes its optical density by dye polymerization process when irradiated. The film develops a distinctive blue color upon exposure to ionizing radiation and becomes progressively darker in proportion to absorbed dose. As Gafchromic films are self-developing, no chemical or physical processing is required after irradiation and the films can be handled in the normal room lights. The film is tissue equivalent, light and flexible and may be cut to desired shape or size. The response of the film is independent of dose rate, dose fractionation and energy above 0.2 MeV.[21] As the main absorption peak of the film is located near 676 nm, the specialized radiochromic densitometers are suitable for sensitive measurements.[22] Film samples of required sizes were prepared 24h in advance to eliminate the artifact due to mechanical compression. Each film sample was given an identification number, which also helped in keeping the same orientation of the film during the readout. Optical density of exposed film was measured using radiochromic densitometer (Nuclear Associates, USA) as well as using Vidar film scanner (Scanditronix/Wellhofer Dosimetrie, Sweden) four days after irradiation. The radiochromic densitometer is a portable manual densitometer which uses a monochromatic light source of peak wavelength 665nm from light-emitting diode. However, Vidar film scanner is a software-controlled imaging device which uses white light and charged coupled device for imaging and OmniPro Accept software for analysis of images.

Prior to each irradiation, TLD-100 (LiF:Mg,Ti) powder (Bicron Technologies Vertriebs Gmbh, Germany) were annealed using a thermal cycle: 400°C (±5°) for 1h - cooling for 6 min - 100°C for 2h and then cooled at normal room temperature. For annealing, the TL powder was placed inside an aluminum foil. Harshaw model 3000A TLD reader was used to record the TL output at maximum acquisition temperature of 290°C using constant heating rate of 10°Cs^−1^. Constant time gap of 24h was maintained between irradiation and readout. Dose response curve for the TLD-100 powder was generated in ^60^Co gamma ray beam (Th-780E, MDS Nordian, Canada) and was found linear in the range of 0.1-1.5Gy.

### Measurement of dosimetry parameters

Three main dosimetry parameters, namely output factor (S_t_), tissue maximum ratio (TMR) and off axis ratio (OAR), which are used for patient dosimetry in SRS/SRT were measured for the three X-Knives. While calculating the magnitude of these dosimetry parameters from their respective experimental values, mathematical relations of Rice *et al*[14] were used.

The output factors of BrainLab cones were measured using CC01, Pinpoint and PP ionization chambers, TLD-100 powder and Gafchromic MD-55-2 films in a full scatter (30 × 30 × 30 cm^3^) PMMA phantom at d_m_. For ionometric measurements of S_t_, reference point of the ion chamber was placed at d_m_ along the central axis of the beam. The chamber was irradiated for 200MU and the corresponding electrometer reading was recorded and the mean of ten readings was used for calculation of output factor for each circular cone. For measurements using TLD, about 30 mg of the freshly annealed TLD-100 powder was packed in square polyethylene pouch. During preparation of the TL pouch, care was taken to keep active length of the pouch smaller than the radius of the circular cone (3 × 3-10 × 10 mm) in all the cases. The TL pouch was placed in the PMMA phantom at d_m_ along the central axis of the beam and was irradiated for 70MU. The TL output of about 5 mg powders was recorded using Harshaw TLD reader and this way six readings were obtained from each TL pouch. The mean value of net TL output per unit weight (nC/mg) of these six readings was used for calculation of output factors of X-Knife circular cones. For radiochromic film measurements of S_t_, samples of size 2 × 2 cm^2^ were placed at d_m_ in the PMMA phantom in such a way that the central axis of the beam was passing through the geometric centre of the film. Each film sample was irradiated for 700MU and the mean of ten optical density values taken from the central region of each exposed film was used for calculating output factor of the circular cone.

S_t_ of Radionics circular cones and mMLC-shaped narrow symmetric square fields were measured using Pinpoint chamber. Equivalent circular diameter (D_eq_) of mMLC square field (S_mMLC_) was calculated using the relation, D_eq_ = 1.122 S_mMLC_.[23] A curve was plotted between S_t_ and D_eq_ and a fourth order polynomial fit (best fit with R^2^ = 0.998) for the data was obtained. S_t_ for desired D_eq_ was calculated using the fitted expression and compared with the output factors of corresponding circular cones.

TMR and OAR of circular cones and mMLC square fields were measured using Pinpoint and CC01 ionization chambers and three-dimensional radiation field analyzers (RFA) available to the hospitals (MP3, PTW, Germany). As the option for direct measurement of TMR was not available to any of the RFA used, percentage depth dose (PDD) was measured and converted to TMR using the conversion software available with the RFA. To determine OAR, beam profiles of 20 mm diameter cones and 18 × 18 mm^2^ (D_eq_ = 20.2 mm) mMLC field were measured at 5.0 cm depth in SAD setup. In these measurements, symmetrical axis of the chamber was perpendicular to the beam axis and during OAR measurement the chamber was moved perpendicular to its axis of symmetry.

## Results and Discussion

### Output factor

**Figure 1:**
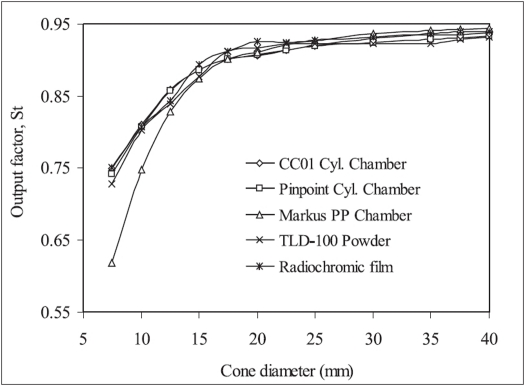
Comparison of output factors of BrainLab radiosurgery circular cones measured using various detectors

Measured output factors of BrainLab circular cones using different detectors are shown in Figure 1. On comparison of the experimental data we observe that S_t_ measured using CC01 and Pinpoint ionization chambers are in excellent agreement with each other for all the cones. TLD and radiochromic film measured output factors are also in agreement with cylindrical ionization chamber measured values within 3% for all the cones. However, Markus chamber measured data for 7.5 and 10.0 mm diameter cones differ from Pinpoint chamber measured data by 16.6 and 7.3%, respectively. This indicates that the Markus chamber is not suitable for dosimetry of circular cones smaller than 12.5 mm. It is therefore advisable that the mean of Pinpoint and Markus chamber measured S_t_ values for circular cones of diameter smaller than 12.5 mm should not be used for clinical dosimetry.

**Figure 2:**
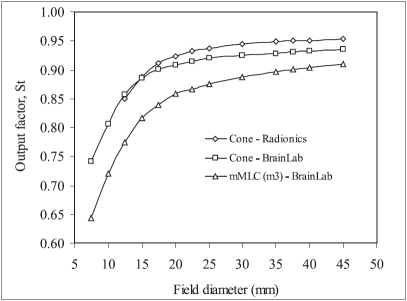
Comparison of output factors of different radiosurgery collimators measured using Pinpoint ionization chamber

Figure 2 shows the variation of output factor with field diameter of Radionics and BrainLab SRS cones and mMLC-shaped narrow fields measured using Pinpoint cylindrical ionization chamber. It is obvious from the curves in this figure that output factor of small fields show strong field size dependence with rapidly decreasing output factor as the field diameter decreases. This is due to rapidly decreasing primary dose for field sizes smaller than the lateral electron range where lateral electronic equilibrium no longer exists.[6,8] A careful observation of curves in Figure 2 reveals that output factors of BrainLab and Radionics circular cones are nearly equal to each other (within 2%) for the entire field diameter. The slight variation in the output factor of the SRS circular cones from these two manufacturers may be because of the difference in the physical dimensions and material of the cone. It is also observed from this figure that output factors of BrainLab mMLC defined equivalent diameter fields are significantly different from output factors of corresponding BrainLab SRS cones. This difference in output factors of BrainLab mMLC defined fields and SRS cones are field size dependent and decreases with increasing field diameter. Numerically, the difference in output factors of the two SRS systems of BrainLab is in the range of 3-15%. The reason for such a large difference in the output factor of two SRS systems from the same manufacturer may be the difference in material and design of the two systems and comparatively high leakage from the mMLC. This observation is contrary to the findings of Cosgrove *et al*.[9] It is notable here that Cosgrove *et al*[9] carried out measurements defining circular fields by mMLC and compared the results with the corresponding circular SRS cones. However, in the present study measurements were carried out with square mMLC fields and equivalent diameters of these square fields were calculated using the relation recommended by Dutreix *et al*.[23] Failure of the recommended relation in case of narrow fields to give exact equivalent diameter of mMLC-shaped square fields may be another reason for such a vast difference in output factors of equivalent diameter mMLC fields and corresponding circular cones. Experimentally determined output factors of BrainLab mMLC defined circular fields (diameter ≥12 mm)were found in agreement with BrainLab circular cones within 6% which further supports the above explanations.

User-measured output factors of four different X-Knives, which use m3 mMLC as tertiary SRS/SRT collimator and Siemens Primus, Varian Clinac 2100 CD and Varian Clinac 2300 CD medical linear accelerators respectively are shown in Table 2. It is observed from the data in this table that output factors of these four mMLC are within 3% for all field sizes except 6×6mm^2^ field where the difference goes up to 8%.

**Table 2:** Variation of output factors of mMLC (m3) with the make and model of the medical linear accelerator

*Square field size (mm^2^)*	Output factor of mMLC (m3) installed at			

	*Siemens primus (H1)*	*Siemens primus (H2)*	*Varian clinac 2100 CD*	*Varian clinac 2300 CD*
6 × 6	0.612	0.621	0.661	0.650
12 × 12	0.800	0.808	0.786	0.810
18 × 18	0.859	0.869	0.847	0.865
24 × 24	0.881	0.889	0.874	0.889
30 × 30	0.895	0.904	0.884	0.906
36 × 36	0.904	0.916	0.906	0.913
42 × 42	0.913	0.925	0.917	0.925

### TMR and OAR

**Figure 3:**
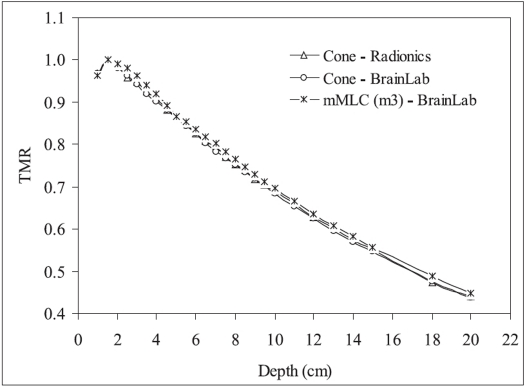
TMR of radiosurgery circular cones of diameter 20 mm and 18 × 18 mm^2^ (D_eq_ = 20.3 mm) mMLC field measured using Pinpoint ionization chamber

**Figure 4:**
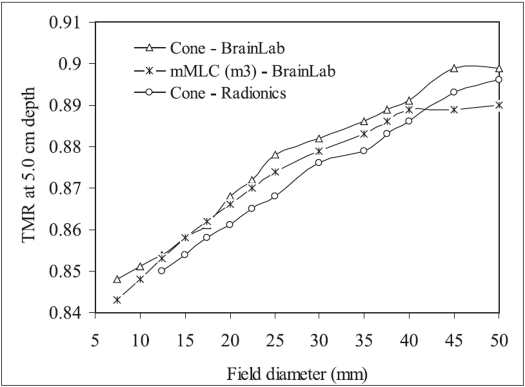
Tissue maximum ratio (TMR) at 5.0 cm depth of radiosurgery circular cones and mMLC defined fields measured using Pinpoint ionization chamber

Figure 3 shows the measured TMR of 20 mm diameter circular cones and 18×18 mm^2^ mMLC field (equivalent diameter = 20.3 mm). From this figure it is observed that TMR of these SRS systems are very close to one another (within 2%). Figure 4 shows the variation of TMR at 5.0 cm depth with diameter of SRS circular cones and mMLC field size. From this figure it is observed that TMR increases with increasing cone / field diameter for the three SRS/SRT systems. TMR of mMLC field shows the tendency of saturation for field sizes greater than 40 mm. The increase in TMR with cone / field diameter is due to increased contribution of scatter radiation with increasing cone/ field diameter. However, the tendency of saturation for mMLC field may be due to the combined effect of leakage radiation and limited range of scattered radiation. It is also observed from this figure that TMR at 5.0 cm depth of the three radiosurgery systems are very close to one another.

**Figure 5:**
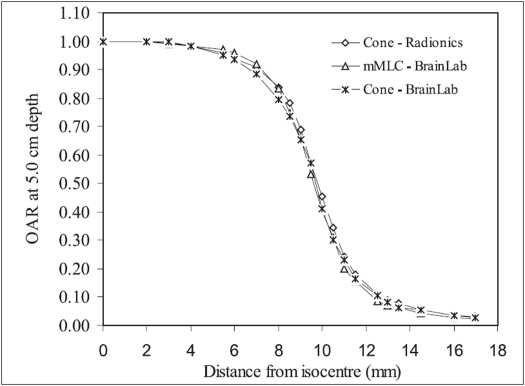
OAR of circular radiosurgery cones of diameter 20 mm and 18 × 18 mm^2^ (D_eq_ = 20.3 mm) mMLC field measured using Pinpoint ionization chamber

Figure 5 shows the variation of OAR at 5.0 cm depth of 20 mm diameter circular cones and 18 × 18 mm^2^ (D_eq_ = 20.3 mm) mMLC field with distance from the isocentre. Because of symmetric nature of the beam profiles, only half profiles are shown here. The figure indicates that the OAR for Radionics cone and BrainLab mMLC fields are very close to each other (within 2%) at all the distances from the isocentre. The OAR of BrainLab cone is comparable to OARs of Radionics cone and BrainLab mMLC within maximum variation of 4%. The widths of plateau (OAR ≥0.97) for these three SRS/SRT systems are very close to one another. The small difference in OAR of BrainLab and Radionics cones and BrainLab mMLC may be due to difference in design aspects and material of construction of the three radiosurgery systems compared here. Further, OARs of 20 mm diameter circular cones and 20 mm diameter mMLC circular field were also found comparable to one another in similar fashion as described above with a minor spatial variation in OAR of mMLC field.

User-measured TMR and OAR of other two X-Knives, which uses m3 mMLC as tertiary SRS/SRT collimator and Varian 2100 CD and 2300 CD medical Linac, respectively were also compared with corresponding TMR and OAR measured on Siemens Primus Linac and found comparable to one another.

## Conclusions

Measurements of output factors were carried out using three different ionization chambers, TLD and radiochromic film to verify the suitability of smallest volume CC01 ionization chamber. Measured data indicated that Compact (CC01) and Pinpoint ionization chambers give approximately the same values. So, further miniaturization of ionization chambers for dosimetry of very small fields may not be helpful as such fields have the inherent characteristics of lateral charged particle disequilibrium. Markus PP chamber was found unsuitable for dosimetry of SRS fields smaller than 12.5 mm. So, the practice of measuring output factors of SRS fields using Markus PP chamber and miniature cylindrical ionization chamber and using the average values of these two measurements for clinical dosimetry give erroneous dose values.

Measurements were also carried out on three different X-Knives to compare their dosimetric characteristics. Output factor of BrainLab and Radionics circular cones were found very close to each other while output factor of mMLC-defined equivalent fields were observed significantly different from circular cones particularly for field diameter smaller than 12.5 mm. TMR of Circular cones and mMLC fields were very close to one another. OARs of Radionics cones and BrainLab mMLC fields were found very close to each other (within 2%) while OARs of BrainLab cones were comparable to OARs of Radionics cones and mMLC fields within a maximum variation of 4%. The concept of switching over to mMLC-based SRS/SRT is thus validated from dosimetric characteristics as well.
